# NK cells and poxvirus infection

**DOI:** 10.3389/fimmu.2013.00007

**Published:** 2013-01-28

**Authors:** Deborah N. Burshtyn

**Affiliations:** Department of Microbiology and Immunology, University of AlbertaEdmonton, AB, Canada

**Keywords:** natural killer cells, poxvirus, ectromelia virus, vaccinia virus, NK receptor, MHC class I, Qa-1, NKRP1

## Abstract

In recent years, our understanding of the role of natural killer (NK) cells in the response to viral infection has grown rapidly. Not only do we realize viruses have many immune-evasion strategies to escape NK cell responses, but that stimulation of NK cell subsets during an antiviral response occurs through receptors seemingly geared directly at viral products and that NK cells can provide a memory response to viral pathogens. Tremendous knowledge has been gained in this area through the study of herpes viruses, but appreciation for the significance of NK cells in the response to other types of viral infections is growing. The function of NK cells in defense against poxviruses has emerged over several decades beginning with the early seminal studies showing the role of NK cells and the NK gene complex in susceptibility of mouse strains to ectromelia, a poxvirus pathogen of mice. More recently, greater understanding has emerged of the molecular details of the response. Given that human diseases caused by poxviruses can be as lethal as smallpox or as benign as Molluscum contagiosum, and that vaccinia virus, the prototypic member of the pox family, persists as a mainstay of vaccine design and has potential as an oncolytic virus for tumor therapy, further research in this area remains important. This review focuses on recent advances in understanding the role of NK cells in the immune response to poxviruses, the receptors involved in activation of NK cells during poxvirus infection, and the viral evasion strategies poxviruses employ to avoid the NK response.

## INTRODUCTION

Natural killer (NK) cells are generally part of the first line of defense to viral infection, but their relative importance to the immune response can vary depending on the virus (Bukowski et al., 1983; Lanier, 2008). NK cells can limit viral replication directly by lysing virus-infected cells, and are also stimulated during a viral infection through cytokines to produce antiviral cytokines such as interferon-gamma (IFN-γ). NK cell responses are rapid and rely on germ-line encoded receptors systems to detect infection, leading to their categorization as innate immune cells. However, NK cells are lymphocytes by lineage and do share features with the adaptive immune response. Importantly, as first documented in mice with murine cytomegalovirus, cognate stimulation through a “pathogen-specific” receptor (Ly49H) expressed by a subset of NK cells, leads to specific proliferation and maintenance of populations of NK cells with heightened function in a form now generally accepted as NK cell memory (Arase et al., 2002; Sun et al., 2009). While the role of NK cells during herpes virus infection has been well established and dissected, poxviruses also present an interesting family of viruses to consider in the context of the NK cell response.

## BASIC BACKGROUND ON POXVIRUSES

Suggestive of their ancient origin, poxviruses infect a wide variety of forms of life with families of poxviruses that infect invertebrates, reptiles, birds, and mammals. Poxviruses that infect mammals are further divided into a number of subgroups including the well-studied orthopox genus. The orthopox genus includes many different viruses that are both medically relevant and well studied as models for pathogenesis such as: ectromelia virus, a natural pathogen of mice; variola virus, the causative agent of smallpox; cowpox, a virus that is maintained in rodents but able to infect a broad range of mammals; and vaccinia virus, a virus likely derived from an extinct equine virus. Vaccinia virus was used as the vaccine successfully to eradicate variola virus and remains under investigation as a general vaccine vector as well as an oncolytic virus. Orthopox viruses cause a wide range of diseases and even a single type of virus will have a range of clinical course in different individuals. For example, while variola virus caused only a mild and localized disease in some individuals, many succumbed to severe disease with very high mortality rates especially for some strains of the virus (reviewed in Bray and Buller, 2004). A much lesser known poxvirus that infects only humans is Molluscum contagiosum that resides in a genus of its own and for the most part it causes only a very localized and mild infection. The severity of disease by poxviruses is correlated with host control of viral replication within the incubation phase (e.g., 10–14 days for variola virus; Bray and Buller, 2004). Therefore, while many variables may play into how a particular individual responds to infection, including the dose and route of the inoculum, genetic differences related to the NK cell responses may influence the response to a poxvirus infection in many species including humans.

How the host cell detects the virus within itself and the effects of the virus of cellular proteins may impact the types of molecules cells express or loose that relate to NK cell recognition of an infected cell and the types of cytokines produced that can activate NK cells. Therefore, there are several features of poxviruses that are worth highlighting here to appreciate how NK cells may combat these viruses, particularly in contrast to the situation with herpes viruses. Similar to herpes viruses, poxviruses prevent host protein expression mainly through destruction of cellular mRNA such that some cell surface proteins must diminish over the course of infection through their natural turnover rates. In contrast to herpes viruses, poxviruses generally do not establish persistent or latent infections and therefore may not require the same type of evasion of T cells and only a few poxviruses are known to actively target major histocompatibility complex class I (MHC-I) proteins. However, poxviruses do devote a considerable proportion (~50%) of the their rather large genomes (~200 genes) manipulating the hosts’ innate and adaptive immune defenses (reviewed in Vermi et al., 2011). The need to encode so many immunomodulatory genes has been suggested to stem from the virus’ relative inability to rapidly mutate to evade the adaptive immune response. Of note, cowpox possesses the most genes predicted to modulate the immune system and is thought to be closest to the most ancient orthopoxvirus. The remainder of this review examines the NK cell response to poxvirus infection, current knowledge regarding NK cell recognition of cells infected by poxviruses and various immune-evasion strategies encoded by poxviruses that may influence the NK response.

## THE *IN VIVO* MOUSE NK CELL RESPONSE TO POXVIRUS INFECTION

The majority of what we know about the role of the NK cell response to poxviruses has been drawn from vaccinia virus and ectromelia virus infection of mice which have many similarities and some differences. Many years ago it was recognized that a few inbred strains of mice such as C57BL/6 and AKR/J mice exhibit much greater resistance to ectromelia virus than most inbred strains such as BALB/c and DBA, for which infection is lethal (Briody et al., 1956). NK cells are important to the response to both ectromelia and vaccinia virus as depletion of NK cells in C57BL/6 mice leads to severe infection with ectromelia, and also causes increases in titers of vaccinia virus (Bukowski et al., 1983; Jacoby et al., 1989; Delano and Brownstein, 1995; Fang et al., 2008). At least one immune-evasion tactic of both these viruses is through interfering with the NK cell response using proteins that antagonize IL-18 function (Born et al., 2000; Reading and Smith, 2003). The ectromelia protein SPI-2 and the vaccinia virus protein N1 also act to limit NK cell responses although the mechanism of action is not clear for either of these proteins (Jacobs et al., 2008; Melo-Silva et al., 2011). It is important to mention here that while NK cells are essential for resistance to ectromelia virus, they are not sufficient, and recovery from infection requires antibody as well as CD4 and CD8 T cell responses (Karupiah et al., 1993; Fang and Sigal, 2005; Parker et al., 2007).

Ectromelia infection occurs naturally through abrasions of the footpad and is spreads to organs such as spleen, liver, lung, and even thymus, likely via the lymphatic system. Susceptible mice develop classic pox lesions on the skin and succumb to the infection within days to weeks following infection. When mice are experimentally inoculated in the footpad with a low dose (50 pfu), NK cells are found in increased numbers in the popliteal lymph node 2 days after infection, while the peak in spleen and liver occurs at 6 days (Parker et al., 2007). NK cells are not actively proliferating at these distal sites until day 4–6 and are found surrounding foci of viral production in the liver. Peak NK activity 3–6 days post-infection has been shown in the spleen for several strains including susceptible strains such as BALB/c (Chaudhri et al., 2004). Depletion studies have shown the NK response is required for resistance during the first few days, and by day 5, their depletion does not have a major impact on recovery, likely because the adaptive immune response has taken over (Dennehy et al., 2010). The early viral control appears to be mediated by NK cells recruited to the draining lymph node and perhaps the site of infection in the dermis without proliferation of the NK cells. It is not clear whether the proliferation of the NK cells is required for control in other organs, formation of memory or serves a homeostatic function to preserve NK cell numbers. Although cowpox is actually endemic in wild rodents, a recent report shows that depletion of NK cells in C57BL/6 mice does not affect mortality following footpad inoculation (Pak-Wittel et al., 2013). However, without NK cells replication of the virus in the draining lymph node does increase substantially and the NK cells are recruited to the draining node through a CXCR3 and IFN-γ-dependent process. As will be discussed in detail below, the reason that NK cells do not appear to matter in the response to cowpox in mice may be due to immune-evasion proteins that limit the efficacy of the NK cells.

In contrast to the situation with ectromelia virus, most strains of mice are generally resistant to vaccinia virus infection suffering few symptoms. However, C57BL/6 may control viral replication in the spleen and liver better than C3H mice (Bukowski et al., 1983). Experimental infection of mice is most often done by intraperitoneal injection with relatively high doses of this virus (e.g., 5 × 10^6^ pfu). Older studies show that activated NK cells accumulate in the peritoneal cavity in response chemokines following an intraperitoneal inoculation with vaccinia virus (Natuk and Welsh, 1987; Prlic et al., 2005). Maximal proliferation of NK cells occurs 2 days post-infection in both the spleen and peritoneal cavity, with greater proliferation occurring in the spleen (Prlic et al., 2005). The earlier proliferation in the case of vaccinia virus compared with ectromelia may be a consequence of the time it takes ectromelia to disseminate from the skin to the internal organs where the NK cells will proliferate, but the dose of virus as well as different properties inherent to the two viruses may also impact the NK response. Recently, it was shown that the response to vaccinia virus in mice requires Toll-like receptor (TLR2) signaling directly on the NK cells (Martinez et al., 2010). This is surprising because this study was also the first report of TLR2 on mouse NK cells. Several studies published this past year have illustrated the role of macrophages and related cells in regulating the NK cell response to vaccinia virus following infection IP and through the footpad. Following footpad injection of the highly attenuated modified vaccinia Ankara (MVA) strain, NK cells accumulate in the draining lymph nodes and become activated in a process dependent on CXCR3 and macrophages and type I interferon (Garcia et al., 2012). This virus is captured by subcapsular macrophages in the draining node that prevents its spread to the spleen and these cells are likely important to provide IL-18 to activate NK cells (Kastenmuller et al., 2012). In addition, myeloid-derived suppressor cells are able to regulate NK responses to vaccinia virus and prevent immunopathology through heightened IFN-γ responses as depletion of these cells using anti-Gr1 leads to enhanced NK responses, decreased viral replication but increased mortality following IP injection of wildtype vaccinia virus (Fortin et al., 2012). These authors also showed that granulocytic myeloid-derived suppressor cells could suppress NK activation by vaccinia virus-infected dendritic cells *in vitro*.

A curious feature of the resistance to ectromelia virus is that it wanes quite dramatically with age (Fang et al., 2010). Sigal and colleagues showed that aged mice do not make the necessary cytotoxic T cell responses, but it is the NK and not the T cells themselves that are defective. NK cells from young mice restore the protection from lethal infection by limiting viral spread and the defective NK response is correlated with an intrinsic defect in NK cell migration into draining lymph nodes (Fang et al., 2010). Aged mice can make normal responses to attenuated ectromelia or vaccinia virus suggesting that the action of NK cells at the site of infection early is what is key to containing the viral replication enough to allow the adaptive response to overcome the infection. These observations are puzzling as there is no obvious advantage for a system to allow NK cell function to decline with age in general, and one might expect increases in memory/primed NK cells of conventionally housed animals. However, in evolutionary terms, there might be an advantage to divert energy from general innate immunity to sustaining memory responses to common pathogens in the environment an animal ages. Contrary to the effect of age on resistance to ectromelia in C57BL/6 mice, the lesions produced by vaccinia virus in BALB/c mice decrease with the age of the mice (Tscharke et al., 2002). Recently it was shown that NK cells primed to vaccinia virus are sufficient to protect from a further challenge in the absence of T and B cells (Gillard et al., 2011). The memory NK cells that can mediate the protection are Thy1^+^ and reside in the liver. The significance of the Thy1 marker is unclear, but their presence in the liver is similar to the original description of hapten-primed memory NK cells residing in the liver able to mediate contact-dependent sensitivity reactions (reviewed in Paust and von Andrian, 2011). Along these lines, it would be interesting to determine if NK cells from young mice primed to ectromelia or vaccinia virus can protect aging mice from ectromelia.

There are some interesting older observations that point to the mechanism that NK cells use to prevent lethal disease. Resistance of C57BL/6 mice is more pronounced for the natural route of infection through the footpad or for low doses injected intravenously, while most strains show similar susceptibility to intranasal, intracerebral, or intraperitoneal inoculation (Schell, 1960). Curiously, intradermal inoculation of C57BL/6 mice with vaccinia virus leads to bigger lesions than in BALB/c or CBA mice (Tscharke et al., 2002), but virus titers of the lesions were not performed in this study and therefore the larger lesions could actually be due to immunopathology of a more robust immune response. Perhaps of some significance, the effects on pathogenesis in mice of certain vaccinia virus proteins are only evident when the route of infection is dermal, which may be due to interplay with NK cells that seem to be key to containing virus in the draining lymph nodes. Amongst these is A40R, a protein with some homology to the proteins within the NK gene complex (NKC) such as NKG2A (Tscharke et al., 2002) and the Bcl-2-like protein N1 in vaccinia virus that limits NK cell responses in BALB/c mice (Jacobs et al., 2008). In addition, the importance of NK cells to the response in the skin has also been suggested through studies in a mouse model of the deviated response to vaccinia virus in eczema patients. In a mouse strain that is eczema-prone (NC/Nga mice), local IL-17 limits the NK response in the skin leading to the uncontrolled viral replication and severe erosive lesions similar to the problems associated with vaccinia virus vaccination in humans with eczema (Kawakami et al., 2009).

## ACTIVATING RECEPTORS INVOLVED IN NK CELL RECOGNITION OF POXVIRUS-INFECTED CELLS

**FIGURE 1:**
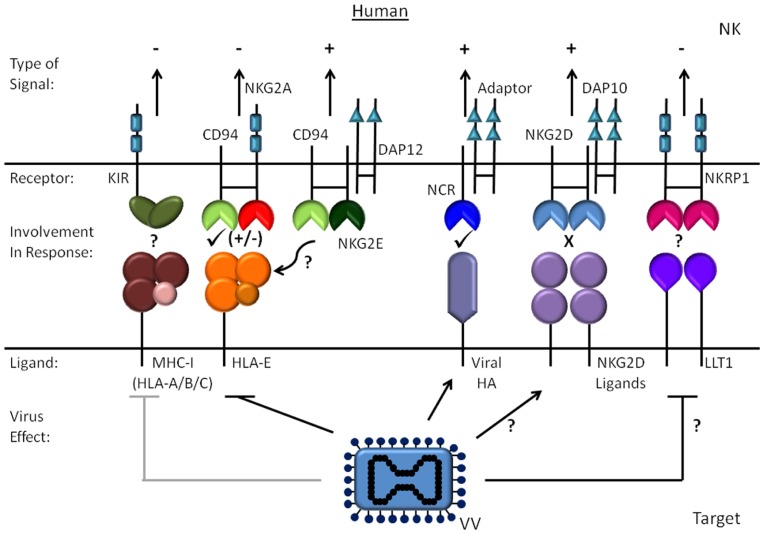
**Human NK cell recognition of vaccinia virus-infected cells.** The activating and inhibitory human receptors and ligands are shown for vaccinia virus infection of human cells. The effect of infection on the ligands is indicated with an arrow for molecules that increase and a line to proteins that are reduced.

Precisely how NK cells become activated during poxvirus infection is not entirely clear and likely has features that are dependent on the virus in question. Given the key role of NK cells in limiting viral replication early and that the control requires both IFN-γ and perforin mediated mechanisms (Fang et al., 2008), several groups have examined the interaction of NK cells with poxvirus-infected cells to determine which activating and inhibitory receptors are important for recognition and cytolysis of an infected target cell. These studies have yielded varying results for vaccinia virus in mouse and human and some apparent differences between ectromelia virus and vaccinia virus. The known activating receptors on NK cells include the natural cytotoxicity receptors (NCR), activating killer cell immunoglobulin-like receptors (KIRs, human) and Ly49s (mouse), NKp65 (human), NKRP1A (mouse), CD94/NKG2C or E, NKG2D and the low affinity Fc-receptor, CD16. To trigger NK cell degranulation, these receptors all couple through small signaling adaptor proteins such as Fcγ, CD3ζ, DAP12, and DAP10. With primary human NK cell lines, the NCRs, NKp30, NKp44, and NKp46, are involved in NK cell lysis of vaccinia virus-infected primary fibroblasts (Chisholm and Reyburn, 2006). In this study, the infected fibroblasts were lysed much more than mock treated cells and this susceptibility required early gene expression from the virus. In this same study, a role for NKG2D in activation was not detected nor any evidence that NKG2D ligands are upregulated as a consequence of infection. Recently, Jarahian et al. (2011) showed that hemagglutinin from either vaccinia or ectromelia virus interact with the human NCRs NKp30 and NKp46. Vaccinia virus hemagglutinin protein is expressed late in infection and counter-intuitively, deletion of the viral hemagglutinin protein from the virus in fact augmented lysis of infected HeLa cells suggesting hemagglutinin interactions with one of the NCRs may actually inhibit NK activation. This group did show that the interaction with hemagglutinin limits NKp30 signaling while weakly enhancing NKp46 signaling, but this was with an NKp30/ζ chimeric receptor which may not behave in the membrane precisely the same as the native receptor. Therefore, it remains unclear what ligands are involved in triggering the NCRs in this context and whether the ligands that induce NK cell activation may depend on the lineage of the infected cell. Taken together, it appears that multiple receptor–ligand pairs govern how a human NK cells are triggered by vaccinia virus-infected target cell (**Figure 1**).

The only NCR expressed by inbred mice is NKp46, and there are no studies directly addressing the role of NKp46 in mouse NK activation by either vaccinia virus or ectromelia virus. However, Sigal and colleagues did show that the signaling adaptor DAP12 and perhaps DAP10 are important for resistance to ectromelia suggesting activating Ly49s and/or NKG2 receptors are involved (Fang et al., 2008). While Ly49H was recently excluded as being required for resistance to ectromelia (Fodil-Cornu et al., 2008), blockade of NKG2D *in vivo* does increase viral dissemination. However, the effect of NKG2D blockade is not as complete as removing NK cells with depleting antibodies (Fang et al., 2008), which may reflect efficiency of blockade or the role of other receptors. In support of NKG2D’s involvement, lysis of target cells infected *in vitro* is partially inhibited by NKG2D blockade. Infection of MEFs also induces a modest increase in binding of soluble NKG2D and expression of the ligands MULT1 and Rae-1 (Fang et al., 2008). At this stage, it is not clear whether the discrepancy in the induction of NKG2D ligands between mouse and human is a difference between how mouse and human cells respond to infection, differences in the cell types studied, or a difference between ectromelia virus and vaccinia virus. As will be discussed in more detail below, more recent studies from the same group showed that CD94/NKG2E is required for resistance to ectromelia virus in C57BL/6 mice (Fang et al., 2011). CD94/NKG2E fits with the requirement for DAP12 in the response to ectromelia because CD94/NKG2E signals through DAP12 (Fang et al., 2011). However, CD94 is not required for the response to vaccinia virus indicating there are significant differences in how NK cells recognize vaccinia virus and ectromelia virus-infected cells (Orr et al., 2010).

## POXVIRUSES AND REGULATION OF MHC-I EXPRESSION AND OTHER INHIBITORY LIGANDS

The current thinking in the field contends that in order for NK cells to respond to infected cells, the barrier of signals emanating from inhibitory receptors has to be overcome. The inhibitory receptors on NK cells that monitor classical MHC-I have shown extremely dynamic and species-specific evolution that was surely shaped by pathogens (Parham, 2008). This diversity includes expansion of distinct receptors such as KIR in primates, cows, domestic cats, dogs, and pigs and Ly49 in rodents and horses. NK cells can also express CD94/NKG2A, an inhibitory receptor that recognizes the non-classical MHC-I protein known as human leukocyte antigen (HLA)-E in human and Qa-1 in mouse. For their own surface expression, HLA-E and Qa-1 require peptides derived from the signal sequences of classical MHC-I proteins, and therefore are believed to serve as a general barometer of MHC-I protein synthesis. It is generally accepted that viruses evolved mechanisms to interfere with MHC-I expression to evade stimulating cytotoxic T cells and then leading to NK cell mediated killing following loss of cell surface MHC-I. The degree to which this mechanism of recognition operates with poxviruses remains unclear and there are only few examples of poxviruses with specific mechanisms known to down-regulate MHC-I. Myxoma virus, a rabbit virus distinct from the orthopox genus, reduces MHC-I expression *in vitro* on cells of various species mediated by a “scrapin” with E3-ligase activity (Guerin et al., 2002; Mansouri et al., 2003). It has not been formally shown that modulation of MHC-I leads to NK cell activation in rabbits, deletion of the scrapin does reduce virulence (Guerin et al., 2002). Cowpox decreases both mouse and human MHC-I protein expression on the cell surface using the protein CPXV203, a protein that sequesters MHC-I from the Golgi back to the endoplasmic reticulum via a KDEL-like motif (Byun et al., 2007; Dasgupta et al., 2007). Given that the reservoir for cowpox is most likely rodents, one might expect it to then encode a decoy protein to target murine MHC-I-specific inhibitory receptors on NK cells to evade NK cell responses. Cowpox does encode an MHC-I-like protein dubbed OMCP for “orthopoxvirus MHC-I-like protein” that was identified by algorithm to predict proteins with an MHC-I fold (Campbell et al., 2007). However, OMCP does not engage MHC-I-specific inhibitory receptors on NK cells. Instead, OMCP is a secreted protein that acts as a high affinity NKG2D antagonist for mouse and human NKG2D (Campbell et al., 2007). Therefore, it appears poxviruses have evolved alternate means to evade activation of NK cells by antagonizing a major receptor for activation as well as antagonizing cytokines involved in NK activation. Interestingly, OMCP also binds to a murine receptor called FcR-like 5, an inhibitory immunoglobulin superfamily protein that is restricted to innate B cells and monocytes/macrophages (Campbell et al., 2010). However, the functional significance of this interaction is unclear as is the normal physiologic function of FcR-like 5 protein. Swinepox (SPV003 and SPV148) and Yatapox viruses also have secreted proteins that have loose homology to MHC-I α chains (Seet et al., 2003) and although they bind to β_2_ microglobulin, they are actually tumor necrosis factor (TNF) inhibitors (Rahman et al., 2009).

It appears then that most orthopox viruses have lost specific mechanisms for disrupting MHC-I expression, and monkeypox is the only other virus with a counterpart to CPXV203. However, a few studies have shown some downregulation of mouse and human MHC-I during infection with vaccinia virus or ectromelia virus. Experiments by one group have shown that human NK cells normally inhibited through CD94/NKGA where the subset of NK cells that lysed vaccinia virus-infected cells suggest that HLA-E is being modulated (Brooks et al., 2006). In these studies the amount of classical MHC-I remained sufficient to provide protection via a KIR (Brooks et al., 2006) however, if HLA-E expression is being compromised it implies production of classical MHC-I is reduced thereby limiting the supply of peptides to stabilize HLA-E. In contrast, we observed changes in MHC-I HLA-C that were sufficient to reduce KIR-mediated inhibition by NK clones (Kirwan et al., 2006). It is likely that the dose of virus and length of infection, as well as the stability of the MHC-I molecules in question influence the degree to which MHC-I loss allows KIR-mediated inhibition to be overcome. It is worth noting though that for human MHC-I, HLA-C is both the major subtype of MHC-I monitored by KIR and is characterized by unstable mRNA and relatively low protein. The question still remains of whether MHC-I modulation has a role in any poxvirus infection *in vivo* and the kinetics of the decrease in MHC-I suggest that for vaccinia virus the reduction is likely due to a non-specific effect of viral disruption of host transcripts occurring later in infection.

The one other poxvirus that is predicted to have an MHC-I homolog is Molluscum contagiosum, a virus in a genus of its own that causes self-limiting skin lesions that can last for months. While the homology of the putative MHC-I homolog MC080R to MHC-I is quite weak, the protein can associate with β_2_ microglobulin (Senkevich and Moss, 1998). MC080R has a predicted transmembrane domain and therefore is unlikely to be an antagonist of NKG2D. In fact, the protein remains in the ER even with vaccinia virus infection, in a manner reminiscent of UL18 from HCMV (Senkevich and Moss, 1998). Unfortunately, challenges in propagating the virus in tissue culture and the lack of an animal model means there is no information regarding the effect of Molluscum contagiosum on MHC-I expression or immune cell recognition of infected cells. None-the-less, it would be interesting to test if the MHC-I-like protein from Molluscum contagiosum binds to any of the MHC-I-specific inhibitory or activating receptors from human. If Molluscum contagiosum could inhibit NK cells, it might fit with how the lesions remain virtually inflammation free for months.

Finally, we examined the possibility that the NKRP-1 receptor system might be important in the response to infection by poxviruses similar to its role in rat CMV infection (Voigt et al., 2007). We found that Clr-b, the ligand of NKRP-1B, is reduced in cells infected by either ectromelia virus or vaccinia virus and that this does limit inhibition mediated through Clr-b (Williams et al., 2012). However, we also observed that when infected these mouse cells were relatively refractory to lysis by IL-2 activated mouse NK cells from CD-1 mice, effector cells that uniformly express NKRP-1B. The reasons for the relatively low lysis of the target cells might be due to other specific or non-specific immune-evasion mechanisms that prevent stimulation [e.g., viral hemagglutinin, decreases in intercellular adhesion molecule (ICAM), evasion of apoptosis cascade, etc.]. Further studies are required to determine if modulation of this set of ligands occurs in other species with the relevant poxviruses and how CD-1 mice deal with ectromelia virus infection. It may also be interesting to consider how other members of this family may play a role in NK cell recognition of pox infection, such as lectin-like transcript-1 (LLT1), which is the human homolog of Clr-b. For humans, the related receptor NKp65 might be of relevance too given the ligand of NKp65 is keratinocyte-associated c-type lectin, a molecule that is selectively expressed by keratinocytes and is perhaps able to stimulate NK cells that are making their way to the epidermis (Spreu et al., 2010). This could be most pertinent for infection with Molluscum contagiosum or dermal infection with vaccinia virus.

## THE GENETIC BASIS OF ECTROMELIA VIRUS RESISTANCE

Many years ago it was noticed that most inbred strains of mice are highly susceptible to ectromelia virus including A/J, DBA/2J, BALB/cByJ, A.By/SnJ, and C3H/HeJ, while resistant strains include 129, C57BL/6, AKR/J, with C57LJ exhibiting intermediate resistance (Wallace and Buller, 1985; Fang et al., 2008). Classical genetic studies also established that one of four loci that confer resistance to ectromelia virus in C57BL/6 maps to the NKC (Delano and Brownstein, 1995). Of the several receptor gene families that are encoded within the mouse NKC that might influence the response to ectromelia virus, CD94 appears to be an important locus mutated in CBA/2J mice. Sigal and colleagues (Fang et al., 2011) noted that the susceptible strain DBA/J2 mice lack CD94 (Vance et al., 2002), while DBA/2, C57BLB/6 and 129 mice have CD94, suggesting CD94 could be involved in the response to ectromelia virus. To test if CD94 could be the resistance gene in CD57BL/6 mice missing in DBA/2J mice, Fang et al. (2011) infected C57BL/6 mice targeted for deletion of CD94 with ectromelia virus. These mice do not have any noticeable defect in NK cell development or function (Orr et al., 2010), but they are highly susceptible to ectromelia infection and the phenotype is rescued by transgenic CD94 (Fang et al., 2011). CD94 forms complexes with the chains NKG2A, NKG2C, or NKG2E. The NKG2A chain confers an inhibitory signal due to immunoreceptor tyrosine-based inhibitory motifs (ITIMs) in the cytoplasmic domain, whereas NKG2C and NKG2E couple to DAP12 and deliver stimulatory signals. The ligand of all of these receptors is Qa-1, the mouse equivalent of HLA-E in humans. Qa-1 expression is maintained through production of the signal sequences from classical MHC-I protein and is typically thought of as required to protect cells from NK cell lysis through engaging CD94/NKG2A, an inhibitory receptor expressed by a large fraction of NK cells. However, staining for Qa-1 was actually increased on L cells infected with ectromelia *in vitro* 4 h post-infection (Fang et al., 2011). While the increase in Qa-1 is likely what leads to stimulation through CD94/NKG2E, why only NKG2E and not NKG2C functions in this capacity remains to be determined. In addition, how Qa-1 is increased and the nature of peptide loaded into the Qa-1 molecules was not examined. The regulation of the NKG2 chains may also be important in the response. While the inhibitory chain NKG2A is expressed in normal mice in a large fraction of cells, the activating chains NKG2C and NKG2E may need to be induced by cytokines in response to infection. The paucity of antibodies specific for NKG2E makes it difficult to assess properly its expression. It should also be noted that while the CD94/NKG2E complex is necessary for resistance to ectromelia on a C57BL/6 background, it is not sufficient to confer resistance on a DBA/2 background as CBA/2NCr mice express CD94 (Brownstein et al., 1991; Vance et al., 2002) and yet are highly susceptible to ectromelia. Therefore, there must be other defects in CBA-derived lines that confer susceptibility to ectromelia. It could be due to another problem within the NKC such as NKG2E, the ligand Qa-1, or a defect in other key pathways such as interferon. Also coincidentally, several of the susceptible strains such as BALB/c have relatively low levels of messenger RNA for three members of the NKRP1 gene family (Giorda et al., 1992).

The role MHC-I in resistance to ectromelia virus (and therefore ultimately Ly49s) was initially thought to be negligible because similar resistance to the virus was found when the H-2^b^ MHC-I genotype of resistant C57BL/6 was compared with H-2^d^ genotype from the susceptible DBA/2J and BALB/c strains on a B10 background (O’Neill et al., 1983). Subsequent crosses of DBA2/J and C57BL/6 did reveal one of the four resistance markers, rmp3, to be on chromosome 17 near the H-2 locus but not H-2D^b^ itself (Brownstein et al., 1992). It is quite possible that the rmp3 locus is Qa-1 itself, although deletion of Qa-1 in CD57BL/6 does not completely recapitulate the loss of CD94 and produces an intermediate phenotype (Fang et al., 2011). On the other hand, for C57BL/6 mice, the presence of Ly49H is somewhat detrimental to the response to ectromelia virus (Fodil-Cornu et al., 2008). *Ly49h*^-/-^ mice on a C57BL/6 background control viral replication in the spleen by about two orders of magnitude better than wildtype mice, particularly when infected at a low dose in the footpad, which is the method by which a role for NK cells is most evident. This somewhat puzzling observation suggests that Ly49H influences either undetermined shifts in the NK receptor repertoire, or competes for signaling by the receptors required for activating NK cells in the context of ectromelia virus infection. It is possible the effect of Ly49H is through limiting DAP12 availability for CD94/NKG2E.

## QUESTIONS REMAINING

**FIGURE 2:**
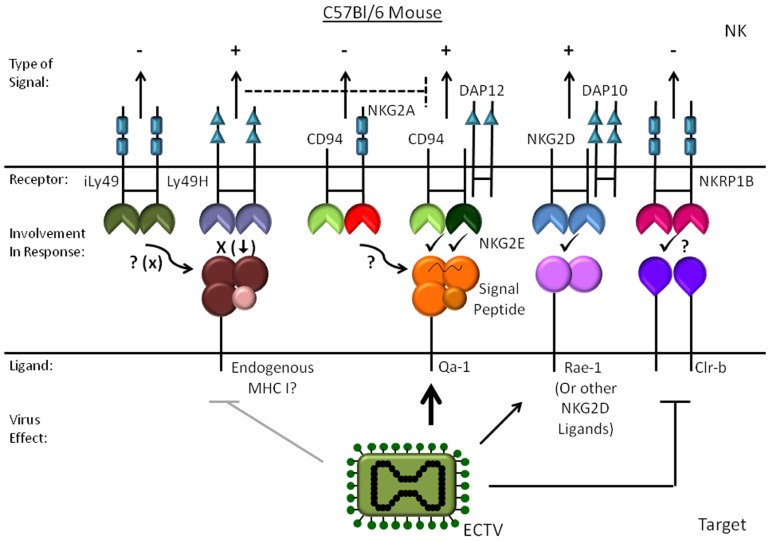
**Mouse NK cell recognition of ectromelia virus-infected cells.** The activating and inhibitory mouse receptors and ligands are shown for ectromelia virus infection. The effect of infection on the ligands is indicated with an arrow for molecules that increase and a line to proteins that are reduced.

In the last few years, many studies have emerged explaining some of the fundamental processes in how NK cells respond to poxviruses and the receptors involved (see **Figures 1 and 2**). Highlighting these is the elucidation of CD94/NKG2E as a key stimulatory receptor required for resistance to ectromelia virus likely in conjunction with NKG2D. Although CD94/NKG2A appears to be necessary for protection from ectromelia virus in C57BL/6 mice, other susceptible strains such as BALB/c express CD94 but are highly susceptible to ectromelia indicating CD94/NKG2E is necessary but not sufficient for resistance. Several interesting questions remain in regard to CD94/NKG2E activation such as what causes the increase in Qa-1, if the peptide loaded into Qa-1 plays a role, and if this receptor plays a role in any other species. Given the homology with NKG2A, it may also be interesting to better understand the mechanism of vaccinia virus A40R that manifests its phenotype only upon dermal infection. Also, since C57BL/6 mice require TLR2 to respond to vaccinia virus, but CD57BL/6 TLR2^-/-^ mice survive ectromelia infection (Sutherland et al., 2011), the ability of vaccinia virus to stimulate TLR2 on NK cells may be a fundamental difference in how mice deal with these two viruses. Other interesting questions include determining how various mouse strains differ in their response to cowpox or monkeypox and the importance of CPXV203 to pathogenesis, and if mouse NKp46 is involved in the response to poxviruses *in vivo*.

It will in the future be interesting to determine if and how these ancient viruses have left an imprint on the NK receptor repertoire of mice or other species, particularly in humans. Although difficult to ever tackle experimentally, variola virus might well have shaped the human NK receptor repertoire given the toll it took on various populations. Molluscum contagiosum presents the opposite extreme in humans as this virus has likely co-evolved with its host to become its current mild and quite persistent form. Genome-wide analyses of the immune response to vaccinia virus in humans have not yet yielded any obvious NK cell-associated proteins. However, one SNP associated with IFN-γ production in T cells in African-Americans (rs1549932), is coincidentally located at 19q13.4, near the region that also encodes the leukocyte receptor complex containing the KIRs (Kennedy et al., 2012a,b).

 A study just published demonstrates that when used as oncolytic virus, poxviruses can prevent post-surgical metastasis by increasing NK activity (Tai et al., 2012). Together, the recent advances suggest better understanding of how NK cells are involved in the immune response to poxviruses in humans is not simply for the sake of curiosity, but should assist rationale design of poxvirus-based vaccine vectors or immune-therapies to elicit the NK response.

## Conflict of Interest Statement

The author declares that the research was conducted in the absence of any commercial or financial relationships that could be construed as a potential conflict of interest.
